# Cofactor-independent RNA editing by a synthetic S-type PPR protein

**DOI:** 10.1093/synbio/ysab034

**Published:** 2021-12-23

**Authors:** Kalia Bernath-Levin, Jason Schmidberger, Suvi Honkanen, Bernard Gutmann, Yueming Kelly Sun, Anuradha Pullakhandam, Catherine Colas des Francs-Small, Charles S Bond, Ian Small

**Affiliations:** Australian Research Council Centre of Excellence in Plant Energy Biology, School of Molecular Sciences, The University of Western Australia, Crawley, WA, Australia; School of Molecular Sciences, The University of Western Australia, Crawley, WA, Australia; Australian Research Council Centre of Excellence in Plant Energy Biology, School of Molecular Sciences, The University of Western Australia, Crawley, WA, Australia; Australian Research Council Centre of Excellence in Plant Energy Biology, School of Molecular Sciences, The University of Western Australia, Crawley, WA, Australia; Australian Research Council Centre of Excellence in Plant Energy Biology, School of Molecular Sciences, The University of Western Australia, Crawley, WA, Australia; School of Molecular Sciences, The University of Western Australia, Crawley, WA, Australia; Australian Research Council Centre of Excellence in Plant Energy Biology, School of Molecular Sciences, The University of Western Australia, Crawley, WA, Australia; School of Molecular Sciences, The University of Western Australia, Crawley, WA, Australia; Australian Research Council Centre of Excellence in Plant Energy Biology, School of Molecular Sciences, The University of Western Australia, Crawley, WA, Australia

**Keywords:** RNA editing, PPR proteins, DYW domain

## Abstract

Pentatricopeptide repeat (PPR) proteins are RNA-binding proteins that are attractive tools for RNA processing in synthetic biology applications given their modular structure and ease of design. Several distinct types of motifs have been described from natural PPR proteins, but almost all work so far with synthetic PPR proteins has focused on the most widespread P-type motifs. We have investigated synthetic PPR proteins based on tandem repeats of the more compact S-type PPR motif found in plant organellar RNA editing factors and particularly prevalent in the lycophyte *Selaginella*. With the aid of a novel plate-based screening method, we show that synthetic S-type PPR proteins are easy to design and bind with high affinity and specificity and are functional in a wide range of pH, salt and temperature conditions. We find that they outperform a synthetic P-type PPR scaffold in many situations. We designed an S-type editing factor to edit an RNA target in *E. coli* and demonstrate that it edits effectively without requiring any additional cofactors to be added to the system. These qualities make S-type PPR scaffolds ideal for developing new RNA processing tools.

## 1. Introduction

RNA processing is an essential and highly regulated step in gene expression in all organisms. Naturally, therefore, there is a lot of interest in developing biotechnological tools that allow intervention in these processing steps to promote or repress the expressions of specific genes. A wide range of different RNA-binding proteins have been investigated for their usefulness in these types of approaches (1–3), but recent work has focused on two particularly promising groups of proteins. Most effort has gone into the exploration of the capabilities of RNA-guided nucleases such as Cas13 (4), but although they have major advantages in the ease with which they can be re-targeted to different RNA sequences, their dependence on their guide RNA cofactors complicates their use in some situations (e.g. if action in a specific subcellular location is required (5)). The second group of proteins that have attracted interest are RNA-binding proteins consisting of tandem repeats that each recognize a single nucleotide. This modular structure facilitates the design of novel synthetic proteins with the desired specificity if the basis for sequence recognition is well understood (6). Several unrelated RNA-binding protein families have converged on a superficially similar alpha solenoid structure where each repeat unit consists of two or three alpha-helices that pack together to form a superhelical surface that can bind single-strand nucleic acid via base-specific hydrogen bonding and stacking interactions (6). Examples of such protein families include the Pumilio/*fem-3* mRNA binding factor (PUF) (7), PPR (8), mitochondrial transcription termination factor (mTERF) (9), octatricopeptide repeat (OPR) (10), heptatricopeptide repeat (HPR) (11) and half-a-tetratricopeptide repeat (HAT) (12) proteins. Of these, the basis for sequence recognition is best understood in the PUF (2, 7) and PPR (13, 14) protein families. PPR proteins consist of tandem repeats of a ∼35 aa helix-turn-helix motif (8) in which two specific amino acids at the fifth and last (usually 35th) position in the motif determine base recognition (14). The derivation of a ‘code’ describing this base recognition has facilitated the development of synthetic PPR proteins based on motif consensus sequences (13, 15–20) that can be readily designed to target any RNA sequence (21) (or even ssDNA (22)). However, so far, most of these synthetic PPR proteins have used very similar scaffolds based on almost identical consensus sequences established from the most widespread form of PPR motif, the so-called ‘P-type’ motif (23). Other variants of the PPR motif exist, particularly in plant organellar RNA editing factors that generally consist of repeated triplets of P, L (long) and S (short) motifs (23). Although very successful in nature (as judged by the huge numbers of such proteins in most plants (24)), these more complex motif structures have disadvantages for biotechnology as the involvement of three different types of motifs (with potentially differing base recognition abilities) complicates rational design. Furthermore, natural PPR RNA editing factors contain differing numbers of C-terminal PPR-like motifs (25, 26). In a ‘full-length’ protein (presumably the ancestral form of active editing factors), arrays of P1-L1-S1 triplets are followed by six additional motifs in the order P2-L2-S2-E1-E2-DYW, where P2 and L2 are slight but characteristic variants of the typical P1 and L1 motifs, the S2 motif diverges considerably from typical S-type motifs and the E1 and E2 motifs are even more divergent 34 aa PPR-like helix-turn-helix motifs (23). The role of these five additional PPR-like motifs in the protein structure and function (and particularly RNA binding) is rather unclear (20, 23, 27). The DYW domain is the cytidine-deaminase-like catalytic domain required for RNA editing (28). In addition to these ‘full-length’ proteins, many apparently truncated proteins exist, lacking one or more of these C-terminal domains. Some of these ‘truncated’ proteins still act as functional editing factors by association with other proteins carrying the missing domain(s) (29, 30). Adding to this complexity, effective, sequence-specific RNA binding by these RNA editing factors appears to be dependent on proteinaceous cofactors, members of the RIP/MORF family (31–33)—at least for the synthetic variants developed to date (20, 34), which have been primarily based on consensus sequences from flowering plants. However, our recent work showed that there is considerable untapped diversity in the PPR motifs of putative RNA editing factors in early branching land plants, particularly ferns, lycophytes and hornworts (24). These plants lack the RIP/MORF cofactors of flowering plants and indeed editing factors from the moss *Physcomitrium patens* function in bacteria (35) and in vitro (36) in the absence of any plant cofactors. One particular PPR motif variant attracted our attention, the S-type motif found in tandem arrays in the editing factors of the lycophyte *Selaginella* (23, 24). This motif is generally only 31 aa long, thus more compact than a typical P-type motif, occurs naturally in monotypic arrays unlike other S-type motifs that occur in P-L-S triplets and comes from a plant lacking RIP/MORF proteins, so it would be expected to show cofactor-independent RNA binding. We tested the possibility of using this S-type scaffold for synthetic RNA binding proteins and have found that it shows considerable promise.

## 2. Material and methods

### 2.1 Design of a dsnS consensus sequence

**Figure 1.**
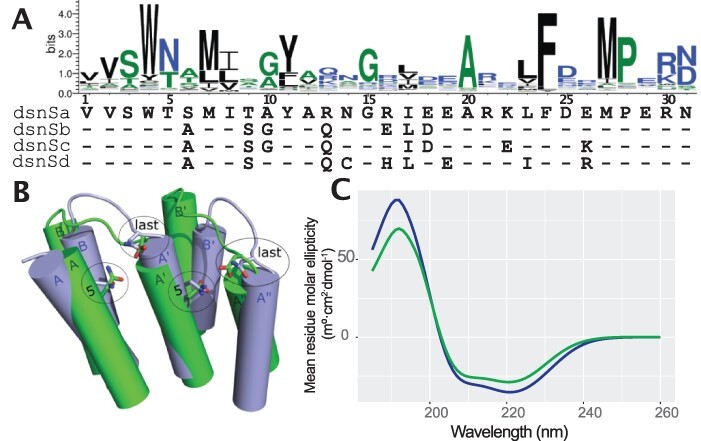
The dsnS-type motif scaffold. (A) Logo graph showing the consensus sequence from the alignment of 4129 natural S-type motifs generated by Weblogo3 (http://weblogo.threeplusone.com/). Below are the sequences of the dsnS scaffolds. (B) An overlay of dsnP (green) and dsnS (blue) motif arrays (2.5 motifs each) modeled by contact site prediction (23) with the fifth and last amino acids of each motif highlighted in stick format (oxygen, red; nitrogen, blue). (C) An overlay of circular dichroism analyses of TRX-9S-S2 (blue) and TRX-9P-S2 motifs (green).

Four designed synthetic S (dsnS) motifs (dsnSa, dsnSb, dsnSc and dsnSd) were used in this study (Figure 1A). The designs were based on consensus sequences from alignments of 31 amino-acid S motifs (dsnSa: 663 sequences, dsnSb: 1101 sequences, dsnSc: 3026 sequences and dsnSd: 225 sequences) identified in 37 land plant species as previously described (23). Each consensus sequence was calculated from the resulting alignment by using EMBOSS cons (37) with the plurality variable set to 0. The same approach was followed to design consensus P2, L2 and S2 motifs that follow S-type arrays in natural RNA editing factors (153 motifs and 35 aa length for P2; 173 motifs and 36 aa for L2; 171 motifs and 32 aa for S2). The P2-L2-S2 domain ends with an 18 aa sequence corresponding to a partial E1 motif based on natural proteins whose last identifiable motif is an S2 motif. Thirty-four C-terminal extensions from such proteins were aligned by MUSCLE (38) and trimmed using trimAl (gt 0.2, cons 20) (39). The most abundant amino acid at each position was then selected to design the C-terminal extension. The P2-L2-S2-E1-E2-DYW domain used in this study was designed in (20). To design the C-terminal solvating helix ending the (dsnSc)_9_ PPR array, the hydrophobic residues in the first helix of the S motif were replaced by hydrophilic residues. All the sequences used for the design of the PPR proteins can be found in Supplementary Table S1. Annotated sequences of the constructs in GenBank format are provided in the online supplement.

### 2.2 Cloning of dsnPPR protein constructs

The various dsnPPR constructs tested in this study are listed in Table 1. DNA sequences (Supplementary Table S1) encoding the 9S-S2, 9S and 9S-DYW proteins were synthesized by GenScript, New Jersey, USA (https://www.genscript.com/). The TRX-9P-S2 gene comprised two gBlocks (Supplementary Table S1) synthesized by IDT Singapore (https://www.idtdna.com) and assembled together by Gibson assembly into pETM20 (40). The Gibson reaction was prepared by mixing 25 fmol pETM20 linearized by NcoI and NotI digestion, 75 fmol of each of the gBlocks and 2✕ Gibson assembly mixture (100 mM Tris-HCl pH 7.5, 10 mM MgCl_2_, 0.2 mM dNTPs, 10 mM DTT, 50 µg/ml PEG-8000, 1 mM NAD^+^, 0.008 U/µl T5 exonuclease (New England Bioabs), 0.05 U/µl Phusion polymerase (New England BioLabs) and 8 U/µl Taq DNA ligase (New England Bioabs)). The reaction was transformed into *E. coli* DH5α competent cells following 30-min incubation at 50°C. The genes encoding the GFP-9S proteins were made in two steps. Initially, we prepared a pETM11 plasmid (40) that has eGFP (Supplementary Table S1) inserted following the N-terminal His tag to create a pET-GFP scaffold. Then, the dsnPPR genes were cloned in frame with the GFP between the NcoI and XhoI or NcoI and NotI restriction sites.

**Table 1.** Constructs used in this research

Construct	N-terminus	Dsn PPR array	C-terminus	RNA target
TRX-9P-S2	TRX-His-	(dsnP)_9_	-P2-L2-S2	*rpoA*
TRX-9S-S2	TRX-His-	dsnSa-dsnSb-(dsnSc)_6-_dsnSd	-P2-L2-S2	*rpoA*
TRX-9S-E2	TRX-His-	dsnSa-dsnSb-(dsnSc) _6_-dsnSd	-P2-L2-S2-E1-E2	*rpoA*
TRX-9S-DYW	TRX-His-	dsnSa-dsnSb-(dsnSc) _6_-dsnSd	-P2-L2-S2-E1-E2-DYW	*rpoA*
GFP-9S	His-GFP-	(dsnSc)_9_	-Solvating helix	*rpoA*
TRX-AviTag-9S-S2	TRX-AviTag-	(dsnSc)_9_	-P2-L2-S2	*rpoA*

The GFP-9S library used to screen different S motifs for their nucleotide specificity was constructed in two main steps. The first step was the construction of the GFP-9S library in pGEM, and the second step involved transferring the gene into the pET-GFP backbone. Assembly of the library followed a method described in (41). Four gBlocks (Supplementary Figure S1A, Supplementary Table S1) were synthesized (IDT Singapore), each with an amino acid combination at the fifth and last positions to target one of the four RNA nucleotides, i.e. Asn/Asp, Asn/Ser, Thr/Asp and Thr/Asn to target U, C, G and A, respectively. The motifs were added sequentially one after the other to the growing pGEM plasmid by using Type IIS restriction enzymes (BbsI and BsaI) as well as SacI and NcoI restriction enzymes (Supplementary Figure S1). To expedite the assembly of the motifs, we made constructs of assembled motifs in parallel and then assembled them together. For example, we constructed sequentially Motifs 5 + 6 and in parallel also assembled Motifs 7 + 8 in pGEM, amplified Motifs 7 + 8 and used the amplified PCR fragment as the insert for ligation with pGEM-M5-6, thus creating pGEM-M5-8.

To partially randomize nucleotide recognition by Motif 4, a motif with fifth/last codons Asn/Asp was amplified from the pGEM plasmid with a set of four forward primers (in the ratio 12:6:1:1) containing nucleotides NDT, VMA, ATG or TGG at the fifth codon and a set of four reverse primers in the same ratio with the complementary partially degenerate codons at the last codon position of the motif (Supplementary Table S2). The choice of codons and primer ratios was based on the recommendations of (42). The diversified motif was ligated into pGEM-M1-3. The ligation was transformed into DH5α cells, and ∼1500 colonies were scraped and used for miniprep. The resulting plasmid was used as a template to amplify the four motifs (M1–M4_randomiz__ed_) and clone them into a pET-GFP plasmid that already contained Motifs 5–9. The ligation was transformed directly into Rosetta 2(DE3) cells and used for picking colonies into 96 deep well plates for screening of binding. We sequenced a library of 644 clones to identify the fifth/last combination in the fourth PPR motif in each of the randomized constructs. This represents about 1.5 times the potential diversity of the library.

All PCR amplifications were done with PrimeSTAR HS ExTaq DNA polymerase (Takara Bio) according to the manufacturer’s recommendations (1 min at 98°C; 35 cycles of 10 s at 98°C, 20 s at 55°C, 10 s 72°C; 1 min at 72°C).

### 2.3 Protein expression and purification

The proteins were made by transforming the plasmids into Rosetta 2(DE3) cells (Novagen) that were grown overnight at 37°C. This preculture was used to inoculate 0.5 l of a fresh LB media at a ratio of 1:50. The culture was grown to OD_600_ 0.4–0.8 at 37°C, transferred to 16°C and, once the culture had time to cool down, 0.1 mM IPTG was added and the bacteria continued growing at 16°C overnight. After spinning down the bacteria the next day, the pellet was either stored at −80°C or immediately resuspended in lysis buffer (50 mM Tris pH 8, 150 mM NaCl, 1 mM beta-mercaptoethanol, 5% glycerol and 20 mM imidazole) and lysed by sonication. Examples of protein expression for the constructs used in this work are shown in Supplementary Figure S2. For experiments requiring pure protein, the lysate was run on Nickel-NTA resin (Bio-Rad Laboratories) columns under gravity. Following elution from the column, the fraction with the pure protein was dialysed into 50 mM Tris pH 8, 150 mM NaCl, 1 mM beta-mercaptoethanol and 5% glycerol and stored in aliquots at −80°C.

### 2.4 Circular dichroism

Purified proteins were concentrated to 10 mg/ml using 10 kDa M.W.C.O. centrifugal filter (Amicon) in Tris pH 8, 200 mM KCl, 5% glycerol buffer. Proteins were then diluted 100 times in circular dichroism (CD) buffer (10 mM KH_2_PO_4_ pH 7.5, 100 mM KF) to 0.1 mg/ml immediately before CD wavelength scan analysis. CD measurements were performed in triplicate using a JASCO J-810 spectropolarimeter with quartz cuvette of 1 mm path length, 100 millidegree sensitivity, 1 nm data pitch, 100 nm/min scanning speed, 2 s response time, 4 nm bandwidth and three accumulations between 185 and 260 nm at room temperature (24°C).

### 2.5 Isothermal titration calorimetry

A Microcal iTC200 from GE Healthcare was used to perform isothermal titration calorimetry (ITC) analysis. Proteins were diluted to 20 mM into Tris pH 8, 200 mM KCl, 5% glycerol buffer. RNA target ligands featuring the *rpoA* footprint (UUACACGUG, synthesized by IDT, Singapore) were made up in the same buffer to 200 mM. Protein samples were placed in the sample cell (cell volume = 200 µl) and titrated with RNA. Titrations were performed at 25°C with a stirring speed of 1000 rpm. RNA was injected 18 times from a computer-controlled syringe at a volume of 2 µl over 4 s for each injection, with a spacing of 150 s between injections. Only 0.5 µl was injected for the first injection and ignored in analysis to minimize potential errors from preparation. Experimental data were fitted to a theoretical titration curve using the Origin software (version 2002, OriginLab Corporation).

### 2.6 Fluorescence polarization

Protein was diluted to 0, 0.781, 1.6, 3.1, 6.25, 12.5, 25, 50, 100, 200, 400 and 800 nM in Tris pH 8, 200 mM KCl, 5% glycerol buffer. Assays were set up in 96-well microtiter plates to 100 µL reaction volumes. Fluorescein-labeled RNA (AUUACACGUG, IDT) was added to a final concentration of 5 nM. Readings were performed on a CLARIOstar Plus microplate reader (BMG Labtech) 532 ± 25 nm/595 ± 35 nm fitted with a polarization membrane. K_d_s were estimated from the binding curves taking into account the probe concentration (43).

### 2.7 Microscale thermophoresis

Cy5-labeled RNA target (Cy5-rpoA) was added at a final concentration of 20 nM to serial dilutions of the protein made in binding buffer (50 mM Tris pH 8, 200 mM NaCl, 1 mM DTT, 50 μg/ml BSA, 50 μg/ml heparin, 0.05% Tween 20). Following 30 min incubation, we measured binding at 20% LED and 40% laser power using a Monolith NT.115 MicroScale Thermophoresis apparatus (NanoTemper Technologies GmbH). The measurements were repeated three times. K_d_s were estimated from the binding curves taking into account the probe concentration (43).

### 2.8 High-throughput RNA pulldown

The His-tagged proteins were diluted in the dialysis buffer and bound to 5 µl magnetic Ni-NTA sepharose beads (GE Healthcare) in a polypropylene PCR plate. After binding, the beads were washed with binding buffer (50 mM Tris pH 8, 150 mM NaCl, 1 mM beta-mercaptoethanol, 50 µg/ml BSA, 3 µg/ml yeast RNA and 0.5% heparin) and resuspended in binding buffer containing 2 nM fluorescently labeled RNA probe. All the washes were done by fixing the beads with a magnet and aspirating the liquid with a pipette. The reaction volume was normally 20 µl. The beads were incubated in the dark between 30 min and an hour and then cooled down on ice for approximately 15 min. The next steps were all done on ice. As quickly as possible, the supernatant of the reaction was removed by a multi-channel pipette, and the pellet was washed in 100-µl ice-cold binding buffer and resuspended in 8 M urea to elute the bound RNA. The eluent was transferred into a fresh PCR plate alongside serial dilutions of the original RNA solution and imaged by a Typhoon scanner (Amersham GE Healthcare) to detect fluorescein (473/>510 nm), Cy5 (635/>665 nm) and IRDye® 800CW (785/814–826 nm). The image was quantified by ImageQuant software (Amersham GE Healthcare). K_d_s were estimated from the binding curves taking into account the probe concentration (43).

Labeled RNA sequences used in this study: Cy5-rpoA = Cy5-AUUACACGUGA; Cy5-rpoA-G = Cy5-AUUAGACGUGA; IR-rpoA-A = IRDye® 800CW-AUUAAACGUGA; IR-rpoA-U = IRDye® 800CW-AUUAUACGUGA; Cy5-clpP = Cy5-CAGCAACAGAAGCCCAAGCUCAUGGA.

### 2.9 Binding screens and analysis

Rosetta 2(DE3) colonies containing the GFP-9S library were picked into 96 deep well plates and grown overnight in 0.5 ml LB at 37°C. This preculture was used to inoculate a fresh 0.5-ml LB following the same protocol as growing culture for protein purification. After the overnight induction at 16°C, the plates were spun and the pellet remaining in the plate was frozen at −80°C (to facilitate cell lysis upon thawing). The cells were resuspended in lysis buffer supplemented with 1 unit of DNaseI (ThermoFisher), 50 mg/ml lysozyme and 0.05% Triton X-100. The resuspended cells were incubated 1 h at 37°C shaking and then spun. The supernatant was used for further analysis. The protein content was quantified by measuring GFP fluorescence from 100 µl lysate and comparing to a standard curve made of dilutions of recombinant GFP purified by nickel affinity column similar to the PPR protein purification and gel filtration (Amersham S200 column using 50 mM Tris and 100 mM NaCl buffer). Unless otherwise specified, 100 µl lysate was used in the screens using the same protocol as for the other RNA-binding measurements. K_d_s were estimated from the binding curves taking into account the probe concentration (43). Estimated K_d_s were capped at 200 nM as, above this, the values were not reliable given the protein concentrations used in the assay. Relative binding strength was estimated by calculating -log(K_d_) and scaling the values between 0 and 1. For comparison with the data from (18), their reported K_d_ values were treated identically (except K_d_s were capped at 900 nM).

### 2.10 *In vivo* editing

The sequence extending 34 bp upstream and 5 bp downstream of the *rpoA* editing site of CLB19 was cloned into the 3ʹ untranslated region (UTR) of the TRX-9S-DYW transcript by amplifying the entire TRX-9S-DYW plasmid using PrimeSTAR HS ExTaq DNA polymerase (Takara Bio) with primers RpoA_GA.For and RpoA_GA.Rev. The resulting PCR fragment was DpnI-digested, gel-purified and circularized using Gibson assembly before transforming into *E. coli* DH5α. The sequence encoding for the amino acids 73–193 of *Arabidopsis thaliana* MORF2 protein was amplified from *Arabidopsis* cDNA using PrimeSTAR HS DNA polymerase (Takara Bio) with primers MORF2_73.For and MORF2_193.Rev (Supplementary Table S2). These primers introduce a C82S mutation as has been done previously to reduce aggregation (20, 34). The resulting PCR fragment was cloned into a linearized pETM11 plasmid backbone (40) between *Nco*I and *Xho*I sites using Gibson assembly and transformed into *E. coli* DH5α.

### 2.11 Quantification of editing and detection of putative off-target editing in the *E. coli* transcriptome

The TRX-9S-DYW and petM11-MORF2 plasmids were sequence-verified and transformed into *E. coli* Rosetta 2(DE3) cells. Five-milliliter starter cultures of Rosetta 2(DE3) were inoculated from single colonies and grown overnight at 37°C at 200 rpm. Two hundred fifty microliter of each overnight culture was inoculated into an Erlenmeyer flask with 25 ml of LB supplemented with the appropriate antibiotics and grown at 37°C 200 rpm until OD_600_ reached 0.4. The cultures were cooled at 4°C for 15 min and then placed at 16°C 180 rpm for a further 15 min. The media was supplemented with 0.4 mM ZnSO_4_ and expression of the PPR and MORF2 proteins was induced by adding IPTG to a final concentration of 0.4 mM. The cultures were grown at 16°C 180 rpm for 20 h before harvesting 1 ml of each culture by centrifugation at 4000 rpm for 2 min. The supernatant was discarded after which the pellets were flash frozen in liquid nitrogen and stored at −80°C until RNA extraction. RNA was extracted from the *E. coli* pellets using the Zymo Direct-Zol RNA miniprep kit (Zymo Research) following the manufacturer’s protocol.

Total RNA extracted as described above was DNase digested using Turbo DNase (Ambion) and quantified using Qubit® (ThermoFisher Scientific, USA). 400 ng of the DNase-digested total RNA was depleted of ribosomal RNA (44) and used for preparing sequencing libraries with the Illumina TruSeq Stranded total RNA library preparation kit as recommended by the manufacturer. The libraries were sequenced on an Illumina HiSeaq4000 sequencer by Novogene. About 30 million 150 nt paired-end reads were obtained for each sample. Reads were first de-duplicated using *clumpify* (using parameters *dedup optical dist = 40*) from the *bbmap* package (https://sourceforge.net/projects/bbmap/), then trimmed of adapters with *bbduk* (parameters: *ktrim = r k = 23 mink = 11 hdist = 1 tpe tbo ftm = 5*) and mapped to the *E. coli* BL21 genome (accession CP010816) and the relevant pETM11 and pETM20 constructs with *bbmap* (parameters: *ambiguous = random mappedonly = t*). Strand-specific nucleotide counts were obtained using in-house code (https://github.com/ian-small/pyrimid). Nucleotide count data was analyzed statistically with a Fisher exact test as implemented in the Python scipy.stats package and the p-values were corrected for multiple testing using statsmodels.stats.multitest.multipletests with the Simes-Hochberg procedure. Odds ratios were calculated after adding a pseudocount of 0.5 to all observations to avoid division by zero.

## 3. Results

### 3.1 The dsnS PPR motif chassis

Similarly to the approach taken to construct synthetic P-type PPR proteins based on consensus motifs, we aligned thousands of S-type motifs extracted from the genome sequences of 37 land plants. Only S-type motifs occurring in monotypic arrays were included in the alignments, meaning that a large proportion (∼32%) of the aligned motifs came from the *Selaginella moellendorffii* genome, where such S-type arrays are common. In these natural PPR proteins, the motifs at the N and C termini of PPR arrays differ slightly in characteristic ways from those toward the center of the arrays. Whether these differences are functionally significant has never been tested. Therefore, from these alignments, we derived four designed synthetic S (dsnS) motifs for different positions in the protein (first motif: dsnSa, second motif: dsnSb, internal: dsnSc and last motif: dsnSd) that imitate the subtle differences between these motifs in native S-type PPR proteins (Figure 1A). In nature, given the average length of PPR arrays, the internal motifs resembling the dsnSc consensus are by far the most abundant.

To compare dsnS and dsnP proteins, we designed nine-motif proteins of each type that target the same RNA sequence and have the same N- and C-termini (Table 1). The target RNA sequence we chose is a nine-nucleotide segment (UUACACGUG) from the *Arabidopsis thaliana* chloroplast *rpoA* transcript. This sequence is part of the binding site for the RNA editing factor CLB19 that has been extensively studied in our lab (20, 45). The synthetic proteins were targeted to this sequence by choosing the amino-acid combination at the 5th and last position of each motif based on the amino-acid code elucidated for P-type proteins (14). As has been experimentally determined for P-class PPR proteins (13, 15, 16), S-type PPR proteins are also predicted to form tracts of α-helical hairpins (23) (Figure 1B), with the base-specific residues (5th and last positions of each repeat) oriented and spaced similarly. This overall structural similarity is supported herein by circular dichroism (CD) analysis of the two proteins, which both strongly display characteristic features of α-helical proteins with maxima at 191 nm and minima at 208 and 220 nm. (Figure 1C).

### 3.2 A dsnS PPR protein binds RNA sequence-specifically and with high affinity

**Figure 2.**
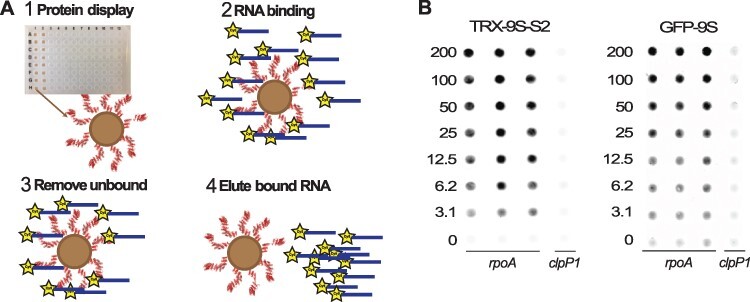
A pulldown assay in a 96-well plate to measure binding. (A) Workflow of the pulldown assay in plates to measure binding. (1) Each well of a 96-well-plate holds a different dilution of a His-tagged PPR protein bound to magnetic Ni-NTA beads. (2) Fluorescent Cy5-labeled RNA is added to each well. (3) After incubation for the desired time, the unbound RNA is washed off. (4) The bound RNA is eluted and quantified using a scanning imager. (B) Scans illustrating fluorescently labeled RNA bound by TRX-9S-S2 or GFP-9S. These proteins differ in their N-terminal and C-terminal tags and in their S motifs (dsnSa-dsnSb-(dsnSc)_6-_dsnSd for TRX-9S-S2, dsnSc_9_ for GFP-9S) but bind similarly strongly to the RNA sequence they were designed to target, Cy5-*rpoA,* and not to a different RNA, Cy5-*clpP1*. The numbers indicate protein concentrations (nM).

To screen for RNA binding activity, we developed an assay in 96-well plates (Figure 2). In this high-throughput RNA pulldown (HRP) assay, the proteins are anchored via a His-tag to magnetic Ni-NTA beads that are dispensed inside the wells of the plate. Each well has a known concentration of protein. Once the proteins are anchored, a fluorescently labeled RNA is added, and after incubation, the unbound RNA is washed off. The bound RNA is eluted and quantified by scanning in a biomolecular imager instrument. Serial dilutions of proteins result in binding curves from which we can calculate the K_d_. Binding measurements from the HRP assay were similar to those made by several different methods (ITC, fluorescence polarization and microscale thermophoresis [MST]) (Supplementary Figures S3, S4, Table 2). The binding affinity of the PPR array does not appear to be greatly affected by the addition of protein tags, as dsnS PPR arrays with different N-terminal fusions all bound similarly (Figure 2, Table 2). Neither did we note any major differences between proteins designed using a single S-motif consensus (dsnSc; TRX-9S-S2) or those designed using all four variants (dsnSa-d; GFP-9S) (Figure 2, Table 2).

**Table 2.** Summary of binding measurements

Construct	TRX-9P-S2		TRX-9S-S2				TRX-9S-E2		GFP-9S	TRX-AviTag-9S-S2
Measurement method	ITC	HRP	FP	ITC	HRP		MST		HRP	HRP
RNA ligand	*rpoA*	*rpoA*	*rpoA*	*rpoA*	*rpoA*	*clpP1*	*rpoA*	*clpP1*	*rpoA*	*rpoA*
Apparent K_d_ (nM)	239	68	7.6	48	6.4	i.d.	6.8	i.d.	12	5.3

i.d. = impossible to determine.

### 3.3 Subtle differences in nucleotide binding specificity between S-type and P-type motifs

Beyond the most common amino-acid combinations at the fifth/last positions in natural PPR proteins, there are many more combinations that occur rarely and which might bind to the four RNA nucleotides with varying affinity and specificity. A few studies have attempted to infer the binding preferences of the various amino-acid-acid combinations from a statistical analysis of the known native PPR proteins and their RNA targets (14, 46–49). Another approach is to survey these combinations experimentally, as was done for P-type proteins by systematically changing the fifth/last amino-acid combinations of two motifs within a 10-motif dsnP PPR protein (18). We have undertaken a similar approach with dsnS proteins to test whether all fifth/last amino-acid combinations work as effectively within both S- and P-type scaffolds and whether they exhibit the same nucleotide specificity.

**Figure 3.**
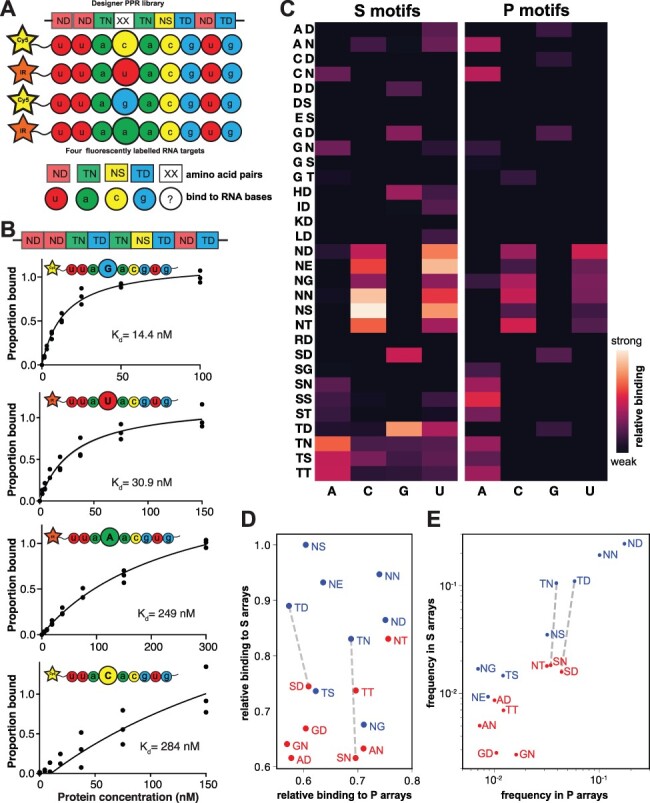
S-type PPR library construction and screening. (A) We constructed a library of dsnS-type proteins each comprising nine motifs. Eight of the motifs are constant, whereas the fourth motif differs in the fifth and last amino acids. We synthesized four RNA targets that are identical in their sequence except in the nucleotide aligning to the variable dsnPPR motif. Each target was labeled either by Cy5 or IRDye® 800C labels. The letters in the box describe the canonical amino-acid combinations and their corresponding target RNA nucleotide in circles. (B) The sensitivity of the library to detect changes in affinity was assessed by measuring the affinities of a single protein variant to four RNA targets that differ in the identity of the RNA nucleotide corresponding to the variable motif. (C) A heat map showing the binding strength of 47 selected variants out of the library to the four nucleotides. The full dataset is in Supplementary Table S3. (D) Relative binding strength differences between dsnS and dsnP motifs. For each fifth/last combination, the binding to the preferred nucleotide has been plotted. The selected combinations include the 16 most common fifth/last combinations in native P-type and S-type PPR proteins. In blue are the combinations relatively more frequent in natural S-type proteins; in red are the combinations relatively more frequent in P-type proteins. P-type data are from (18). Dashed lines connect selected pairs of combinations that recognize the same nucleotide. (E) Relative frequency of fifth/last combinations in natural P-type and S-type proteins.

We designed a library of dsnS proteins targeted to bind the *rpoA* RNA target. All nine motifs were based on the dsnSc consensus. Eight of the motifs were held constant, whilst the fourth motif was a different amino-acid combination in each protein (Figure 3A). To remove any potential influence on binding by the C-terminus, we used a protein scaffold that has a very short C-terminus made of half an S-type helix where the hydrophobic residues were replaced by hydrophilic residues to aid in solvation (GFP-9S, Table 1).

Initially we tested if our assay is sensitive enough to measure changes in affinity that result from differences in the nucleotide alignment of one motif out of nine. To do that, we measured the binding affinities of one dsnS PPR protein (with the fifth/last amino-acid combination TD, predicted to recognize G) to four RNA targets differing only in the nucleotide aligned to the fourth motif (Figure 3B). We found that the oligonucleotide with G at the fourth position was indeed preferred (K_d_ ∼14 nM compared to ∼280 nM for the least favored nucleotide, C). A library was prepared by randomizing the fifth and last codons of the fourth motif (Supplementary Figure S1) using primers designed to reduce redundancy and maximize codon sampling in the library (42). The PPR arrays were constructed as C-terminal fusions to GFP to facilitate quantification of the protein. After verification by sequencing, we found the library contained 254 distinct fifth/last amino acid combinations.

Screening of the library was made easier by bypassing the need to purify the proteins and using the HRP assay to screen bacterial lysates directly. Bacterial growth and lysis were done in deep well plates. PPR protein concentration was quantified in the cleared lysate of each well by measuring the fluorescence of the GFP tag in a plate reader. Serial dilutions of lysates produced consistent binding curves that are close to those produced from purified proteins (Supplementary Figure S5). The various amino-acid combinations greatly affect the binding affinities to the four target RNAs, resulting in a wide range of K_d_s. To be able to assess the full range of K_d_s accurately, we performed the screen three times with five different protein concentrations (Supplementary Figure S6). The resulting measurements could be fitted with binding curves that give estimated K_d_s that can be displayed in a heat map and compared with similarly calculated values from dsnP proteins (18) (Figure 3C, Supplementary Table S3). Binding preferences for the dsnS motifs follow those of dsnP motifs and we found no significant differences in which nucleotides were favored by each fifth/last amino acid combination. However, we did find some clear differences in efficacy. For example, the fifth/last amino-acid combinations TD and SD are equally effective at binding their preferred nucleotide (G) in dsnP proteins, but TD is much more effective than SD in dsnS proteins (Figure 3D). The pairs TN and SN (binding A) show similar differences between dsnP and dsnS contexts (Figure 3D). These differences are unlikely to be simply due to experimental differences in the way the binding was measured for these two types of PPR protein because the same patterns of differences are seen in the frequencies with which these combinations occur in natural PPR proteins. Combinations that bind poorly *in vitro* in a dsnS context (e.g. GN, GD, AN, AD, SN and SD) are also relatively rare in natural S-type proteins (Figure 3E).

### 3.4 RNA binding is relatively insensitive to temperature, pH and salt

**Figure 4.**
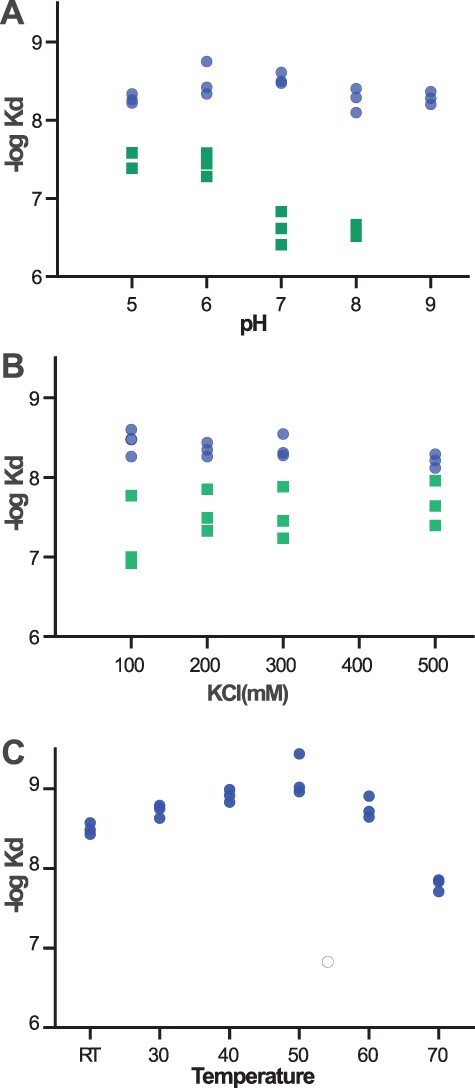
DsnS-type and DsnP-type PPR proteins binding across various pH, salt and temperature ranges. We measured the binding of TRX-9S-S2 (blue spots) or TRX-9P-S2 (green squares) under various conditions using the pulldown plate assay. PPRs were incubated with the target RNA under various pH conditions (A) or salt conditions (B). TRX-AviTag-9S-S2 was incubated with a target RNA under various temperature conditions in a thermocycler (C). The full data for the measurements is in Supplementary Figures S7–S9.

PPR proteins are native plant proteins that are not normally exposed to extreme conditions, however as a synthetic chassis, it could be useful to incorporate dsnS PPRs as a part in synthetic biology systems and circuits that need to operate in various environmental conditions, including high temperatures that destabilize RNA secondary structures. We tested the resilience of the S-type PPR chassis in various pH, salt and temperature conditions. The pulldown assay we developed can easily be used under various buffer and temperature conditions, because the binding step is separate from the fluorescence measurement step, and therefore the extreme binding conditions do not influence the quality of the measurement. We made serial dilutions of purified proteins within the wells of a 96-well plate. The dilutions were made in buffers that had either different pH or different salt concentrations. The washing steps were done with the same buffer as the binding steps. Finally, the bound RNA was eluted in urea and quantified by scanning fluorescence. We found that the S-type proteins bind well under a broad range of salt and pH conditions, unlike our previous P-type chassis which has reduced binding at pH above 6 or in low salt concentrations (Figure 4A and B). The temperature measurement was done similarly, only dilutions were made in PCR-strips that were then incubated with the labeled RNA in a thermocycler. Following the incubation with the RNA at the various temperatures, the beads were washed by diluting them directly into an excess of a buffer that was prewarmed to the binding temperature. The excess buffer was quickly removed and the bound RNA was eluted by urea and quantified. The resulting binding curves show that the S-type proteins are quite stable and functional at high temperatures up to 60°C (Figure 4C).

### 3.5 Site-specific RNA editing in *E. coli* by an S-type PPR protein

**Figure 5.**
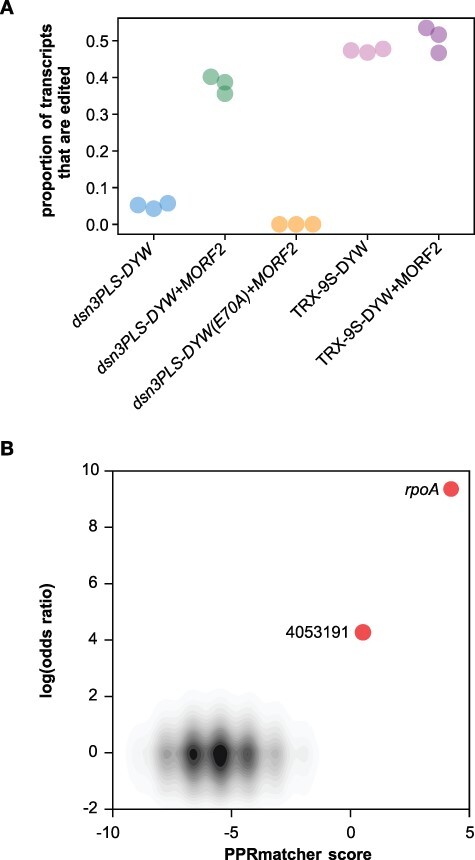
RNA editing in *E. coli* induced by expression of the TRX-9S-DYW editing factor. (A) Proportion of edited RNA-seq reads in samples from bacteria expressing the various constructs. Data on dsn3PLS-DYW constructs are from (20). (B) Potential editing sites plotted by their predicted binding to TRX-9S-DYW (x-axis) against the difference in the rate of editing (expressed as the log of the odds ratio) compared to the negative control (dsn3PLS-DYW(E70A) together with MORF2). Predicted binding scores were calculated with PPRmatcher (https://github.com/ian-small/PPRmatcher) using the Kobayashi scoring table (adapted from 14, 18, 46, 47). Apart from the intended target (*rpoA*), the only significant site where the log(odds ratio) exceeds 2 is the putative off-target editing event at Genome Position 4 053 191. The ∼733 000 sites below this threshold are indicated by the density contours (gray-black).

In plant organelles, S-type PPR motifs are predominantly found in RNA editing factors. This prompted us to design a synthetic RNA editing factor based on an S-type PPR motif array. To test for the ability to edit RNA, we expressed the *rpoA*-targeting S-type PPR array, with additional C-terminal P2-L2-S2-E1-E2 motifs and a DYW domain (TRX-9S-DYW, Table 1). This C-terminal domain has already been shown to function in C-to-U RNA editing in *E. coli* (20). The target RNA editing site was inserted into the 3ʹUTR of the transcript encoding the PPR protein. Following protein expression, we extracted and sequenced the RNA from the bacterial culture. The RNA-seq data analysis showed that the level of editing was about 50% (Figure 5A). RNA editing factors in flowering plant organelles are partially reliant on co-factors (MORF proteins), as was a previous synthetic editing factor we constructed using P, L, and S motifs (20). The expectation was that the S-type PPR array should not be dependent on MORF co-factors, and indeed, we found that co-expression of MORF2 had almost no effect on the amount of RNA editing (Figure 5A). The specificity of the editing reaction was verified by analyzing the RNA-seq data for putative off-target events in the *E. coli* transcriptome. We found a single significant event at Genome Position 4 053 191 in the *tufB* transcript encoding the minor variant of elongation factor Tu (Figure 5B). This editing event alters a proline codon (CCG) to a leucine codon (CUG). The sequence upstream of the editing event (UUGAACGUACAAAAC**C**, with the edited C in bold) is similar to the *rpoA* target site (UUACACGUGCAAAAU**C**), making it a likely off-target. However, the amount of editing is extremely low (14 edited reads out of 10 303), and the identical sequence in *tufA* is edited even less (1 edited read out of 10 419).

## 4. Discussion

In this paper, we present a novel designed PPR protein chassis that we developed based on the alignment of native S-type PPR motifs in a similar approach to that taken previously for P-type proteins (15–18). The chassis is easy to express in large amounts in *E. coli*, is relatively soluble and is predicted to fold into a typical PPR structure composed of tandem helix-turn-helix repeats (Figure 1). We have characterized the performance of dsnS PPR proteins using an RNA pulldown assay we have developed (Figure 2). Many different assays for protein-RNA interactions are available with different advantages and disadvantages. In our assay, the protein is anchored, rather than the RNA. This makes it easier to study protein variants, and by using RNA probes that are labeled with different fluorescent labels, it is possible to simultaneously measure binding to more than one RNA target in the same test. Anchoring the proteins to magnetic beads allows us to introduce washes to the workflow and circumvent the need to purify the proteins. This means we can use proteins directly from cell lysates, greatly facilitating medium- or high-throughput screens. Finally, the screen is extremely sensitive, requiring only small volumes (≤20 µl) and low concentrations of RNA (1–2 nM) allowing the measurement of K_d_s in the nanomolar range. The K_d_s measured by this method were comparable to those obtained by fluorescence anisotropy (and by MST if we consider that the effect of different N- or C-terminal tags are negligible), i.e. 5–12 nM for complexes with the *rpoA* target. The apparent K_d_ measured by ITC was considerably higher (48–68 nM). Whereas the pulldown assay, fluorescence anisotropy and MST are all end-point assays measuring the ‘true’ K_d_ at equilibrium (if the protein/RNA mix has reached equilibrium before the assay), ITC measures the transient enthalpy change when the RNA and protein are mixed. In the case of a complex, slow interaction (likely to be the case for PPR-RNA binding) ITC may reveal the thermodynamics for an initial fast phase of the binding, likely leading to a less stable complex (and thus a higher K_d_) than the final bound state.

The affinity of PPR proteins for their RNA targets is determined by the identity of two amino acids at the fifth and last position of each motif (14). We were able to design a dsnS PPR protein to bind tightly and specifically to its RNA target by using the known amino-acid combinations (14, 18, 46, 47) determined for other PPR proteins. However, in nature, PPR motifs contain a wide diversity of different amino acid combinations, each with the potential to provide a different binding preference. By screening with our HRP assay we were able to measure the binding preferences of nearly two-thirds of all possible amino-acid combinations. The results are highly concordant with those published for dsnP proteins (18), but with some interesting differences. A number of amino acid combinations that are effective and specific in the dsnP context are much less so in the dsnS context. This is particularly noticeable for amino acids with small side-chains (i.e. G, A and S) located at Position 5; these all strongly correlate with purine binding in the dsnP context but not when at the same position of our dsnS motif. Probably as a result of this lack of effectiveness, these amino acids are much rarer at this position in natural S-type proteins than they are in natural P-type proteins. This information will assist in improving the predictions of the RNA targets of uncharacterized natural PPR proteins. Moreover, it improves our ability to design the affinity and specificity of dsnPPR proteins.

To be useful in a wide range of applications, it is essential that dsnS proteins are tolerant to a wide range of physiological (and unphysiological) conditions. In comparison with our previous dsnP chassis, dsnS proteins bind consistently tightly to their RNA target over a broad range of pH, salt and temperature conditions. This makes this chassis a good basis for *ex-vivo* applications and suggests that it should function in a variety of host organisms.

As a demonstration of utility, we constructed a synthetic editing factor using a dsnS PPR array to target a specific cytidine within the UTR of its own transcript. We had previously constructed a similar editing factor using triplet arrays of P, L and S motifs modeled on natural PPR editing factors from plants (20). The previous dsnPLS editing factor (20) required the presence of a plant cofactor protein (MORF2) for full activity and was almost incapable of editing the target RNA on its own (Figure 5A). MORF2 is a member of a small family of similar proteins implicated in organellar RNA editing in plants (31–33), which appear to promote RNA binding by PLS-type PPR proteins through interactions with the L motifs of the PLS arrays (20, 34). The requirement for a cofactor is obviously not desirable for practical applications. Cofactor-independent editing by natural PPR proteins has been reported for editing factors from the moss *Physcomitrium patens* (35, 36). MORF proteins are absent from mosses and other nonseed plants, including lycophytes (24). We hypothesized that dsnS arrays would not require MORF cofactors as they lack L motifs and derive largely from S-type motifs from nonseed plants. This proved to be the case, and the expression of our TRX-9S-DYW editing factor in *E. coli* gave efficient, cofactor-independent editing (Figure 5). The specificity of the RNA editing is excellent, with only a single off-target site detected in the *tufB* transcript, and this had extremely low levels of editing (0.14%). In general, PPR motifs distinguish between purines and pyrimidines much more effectively than they can distinguish between A and G or C and U (18). Of the three differences between the *tufB* sequence and the *rpoA* target (discounting the C/U mismatch adjacent to the editing site which is not in the region bound by the PPR motifs), two are purine mismatches (A/G) and only one is a purine-pyrimidine mismatch (A/C). In addition, the five nucleotides (CAAAA) that would align with the P2-L2-S2-E1-E2 motifs are also identical to those at the equivalent position of the intended target. Based on our prior results with synthetic editing factors (20), this is a plausible off-target event. It is only detectable because of the very large number of reads mapping to the *tufB* transcript.

Targeted RNA editing *in*  *vivo* affords an exciting route to repair or alter the protein content of an organism without permanently changing its genetics (50). In principle, it can be used to correct genetic mutations (51), add diversity to the protein repertoire expressed by a cell (52), provide a novel way to control translation (53) or regulate protein activity (54). Most recent attention has focused on RNA-guided editing enzymes either based on adenosine deaminase acting on RNA (ADAR) directly (e.g. 51) or dCas13-ADAR fusions (e.g. 54). However, the reliance on an RNA cofactor can be a disadvantage in some circumstances (e.g. when targeting organelle transcripts) making protein-only solutions worth exploring. The dsnS arrays described here provide a robust, easy to reprogram, cofactor-independent chassis that can be used potentially for a wide range of RNA processing tools, including RNA editing.

## Supplementary Material

ysab034_Supp

## Data Availability

RNA-seq data are available from NCBI’s Sequence Read Archive under BioProject accession PRJNA680433.

## Material availability

All the constructs listed in Table 1 are available from Addgene (Catalog # 176217–176223).
