# Supplementary material for: Cofactor-independent RNA editing by a synthetic S-type PPR protein
Source: Synth Biol (Oxf). 2021 Dec 23;7(1):ysab034. doi: 10.1093/synbio/ysab034 (PMC8809517; doi:10.1093/synbio/ysab034)
Supplement: ysab034_Supp [file ysab034_supp.zip › Supplementary figures.pdf]

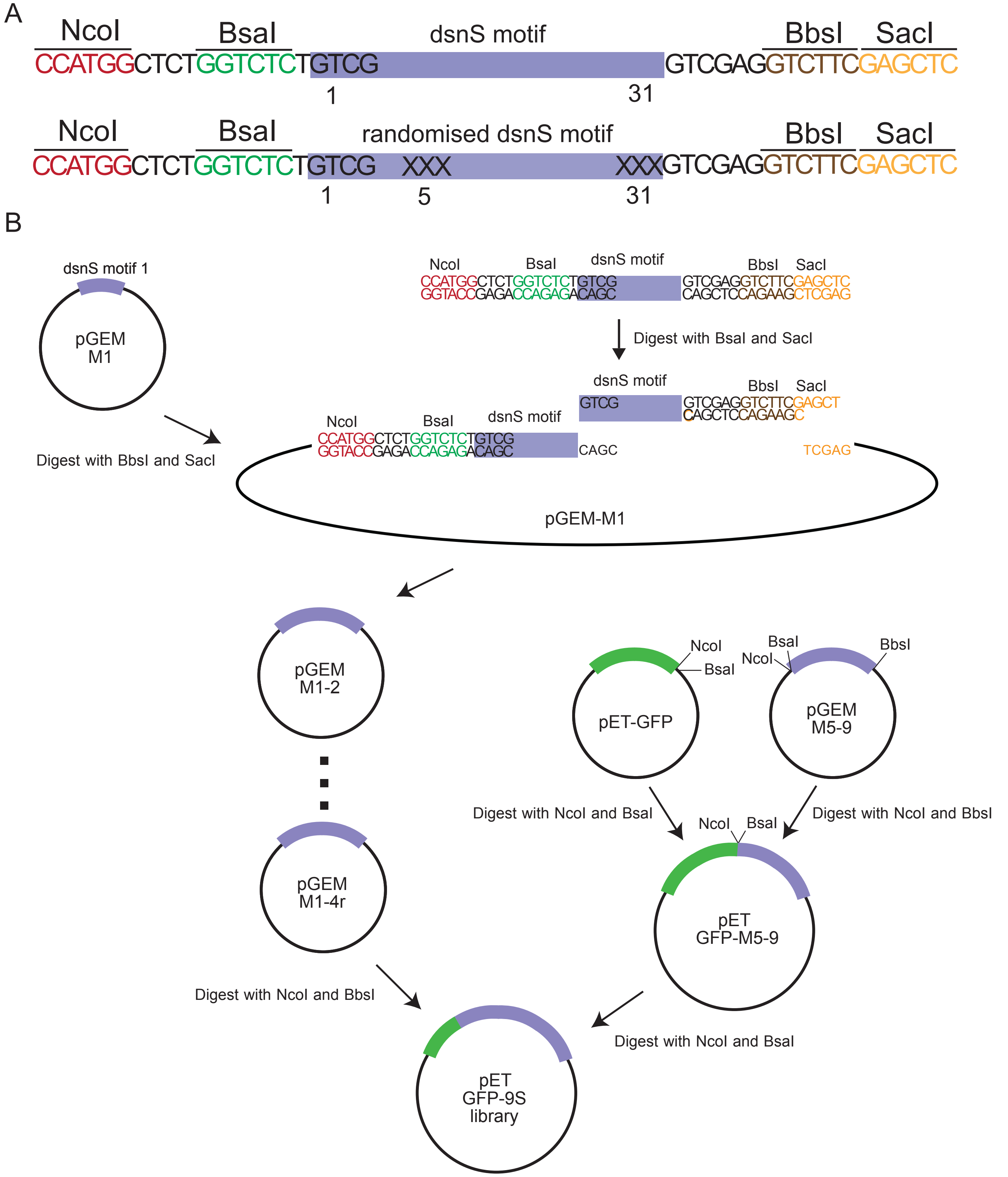

Supplementary Figure S1. Assembly of GFP-9S library by sequential ligation of motifs. (A)The design of a dsnS motif gBlock. (B)The steps in the assembly of the library leading to the final GFP-9S library construct. Motifs were added by ligating gBlocks digested with BsaI and SacI to a pGEM vector opened with BbsI and SacI. Motifs 1-4 (with motif 4 being quasi-randomised at positions 5 and 31 by PCR) were accumulated in one series of ligations whilst motifs 5-9 were accumulated separately. Finally, the S motif arrays were ligated into a pET vector encoding an N-terminal GFP extension.

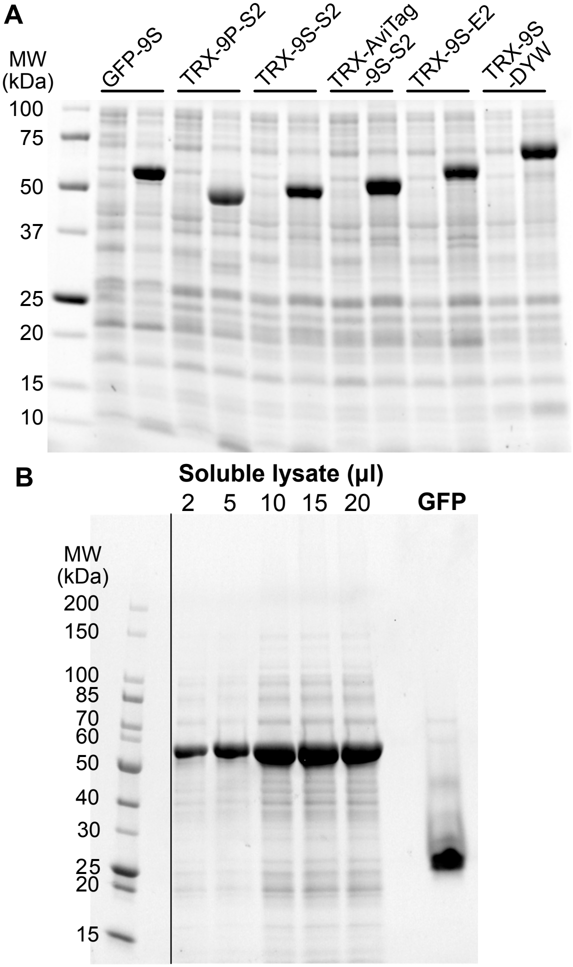

Supplementary Figure S2. Bacterial expression of dsn9S and dsn9P proteins. (**A**) Pairs of pre-induction (left) and post-induction (right) samples for each of the proteins are shown. The expected molecular weights (in kDa) of the expressed proteins are 62.9 (GFP-9S), 63.2 (TRX-9P-S2), 60.5 (TRX-9S-S2), 62.5 (TRX-AviTag-9S-S2), 66.9 (TRX-9S-E2) and 81.4 (TRX-9S-DYW) respectively. Cultures of Rosetta 2 cells were grown at 37°C in a rotary shaker (230 rpm) in LB medium supplemented with 250 mM NaCl, 50 mM TRIS pH 7.5, 1 % glucose and the appropriate antibiotic until the OD_{600} reached 0.7–0.8. The cells were then pelleted and resuspended in the same medium without glucose but with 0.5 mM IPTG to induce expression and grown overnight. For SDS-PAGE analysis, the cells were pelleted and the proteins were acetone-precipitated (600 µL of 0.07 % mercaptoethanol in pure acetone), kept at -20°C for at least 1 hour and pelleted again. Prior to electrophoresis, the samples were dissolved in loading buffer, heated at 65°C for 10 minutes, rapidly spun down and loaded onto a 12 % acrylamide gel. (**B**) Overloading of GFP-9S lysate to check for free GFP. Increasing amounts of GFP-9S lysate was loaded on a 4–20% pre-cast gel (BioRad) with purified eGFP (27 kDa) as a control. Samples were mixed with loading buffer and heated to 95°C for 5 minutes before loading.

TRX-9S-S2

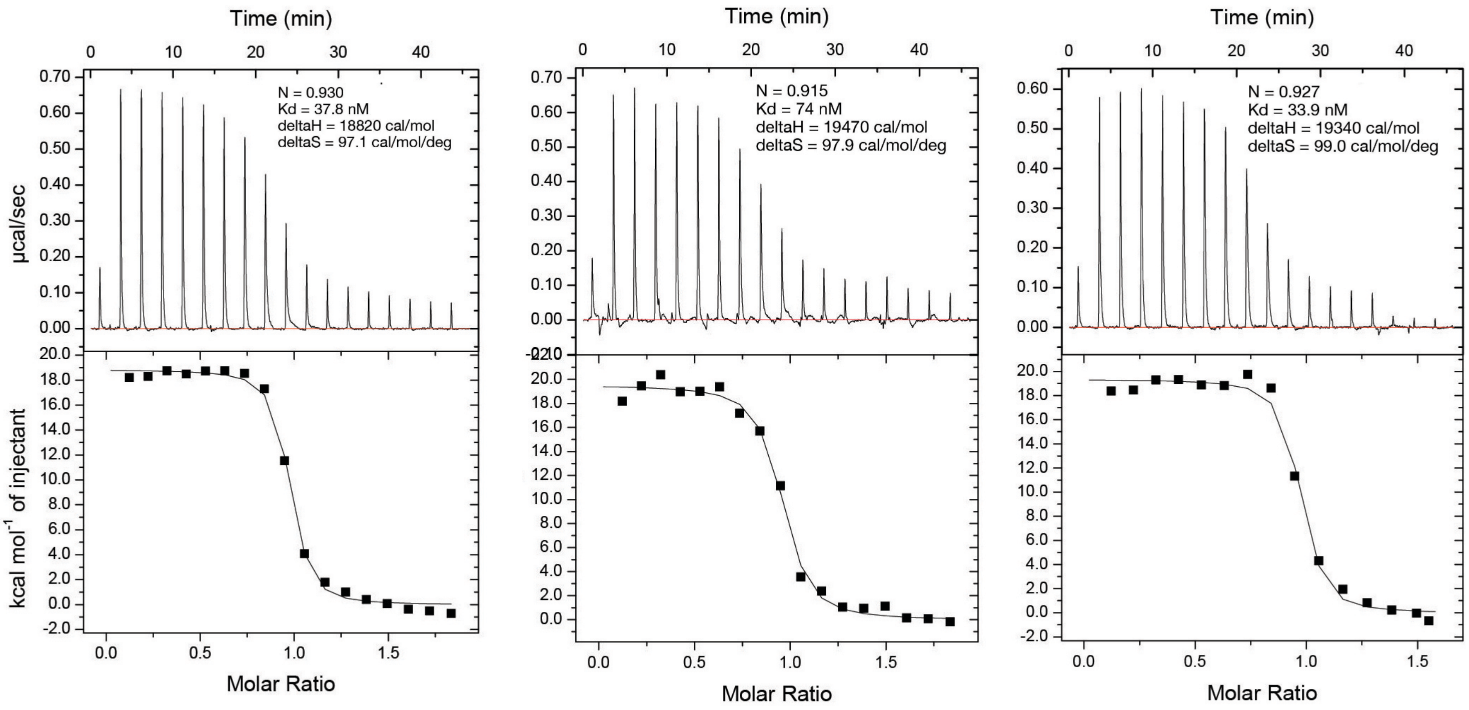

TRX-9P-S2

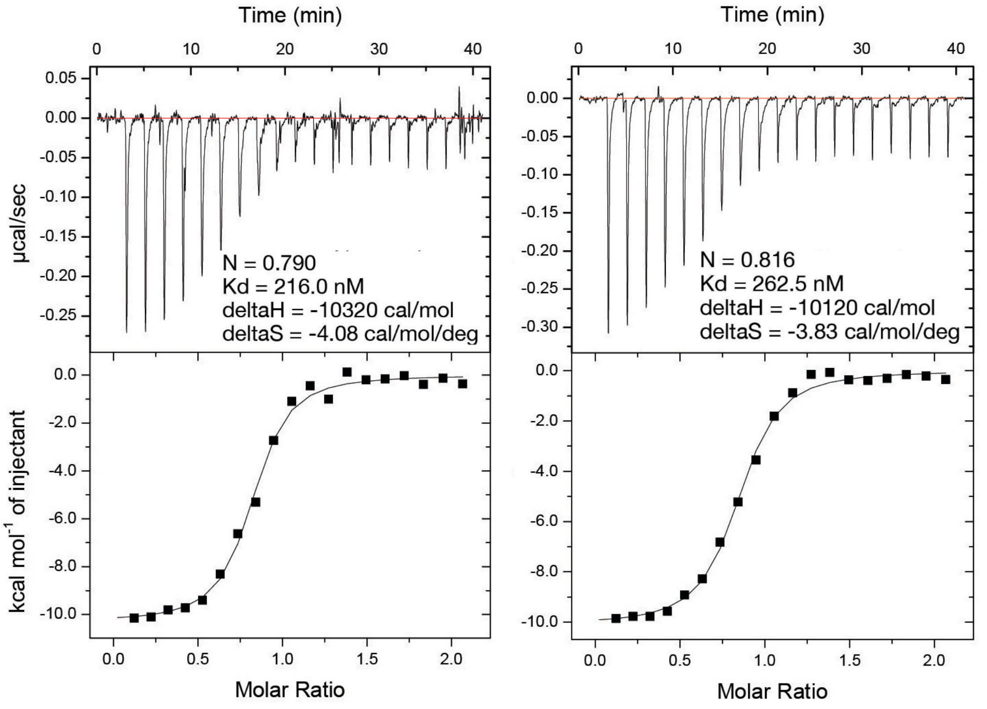

Supplementary Figure S3. Binding of 9S and 9P arrays to rpoA target RNA analysed by isothermal titration calorimetry (ITC). The binding of purified TRX-9S-S2 and TRX-9P-S2 to unlabelled *rpoA* RNA was measured by ITC. The change in heat measured over time (upper panels) was fitted onto a binding curve (lower panels) and used to calculate ΔH, ΔS, N (stoichiometry) and Kd by Origin software. The figure shows three experimental replicates for TRX-9S-S2 and two experimental replicates for TRX-9P-S2.

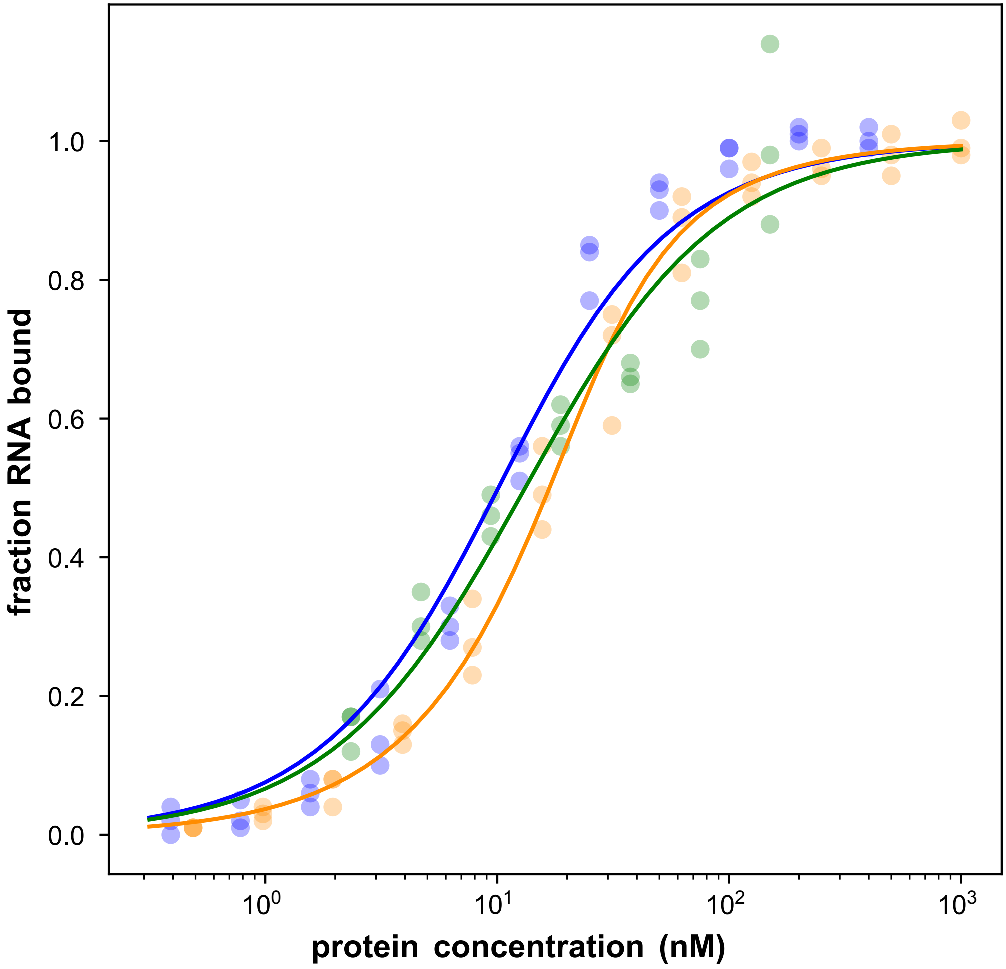

Supplementary Figure S4. Binding of 9S arrays to *rpoA* oligonucleotides measured by fluorescence anisotropy (blue), microscale thermophoresis (orange) or RNA pulldown (green). The proteins tested were TRX-9S-S2, TRX-9S-E2 and GFP-9S, respectively. The apparent K_d values estimated from the fitted curves were 7.6 nM, 6.8 nM and 12 nM respectively. The curve-fit equation used was equation (3) from Pagano et al. RNA (2011), 17:14–20.

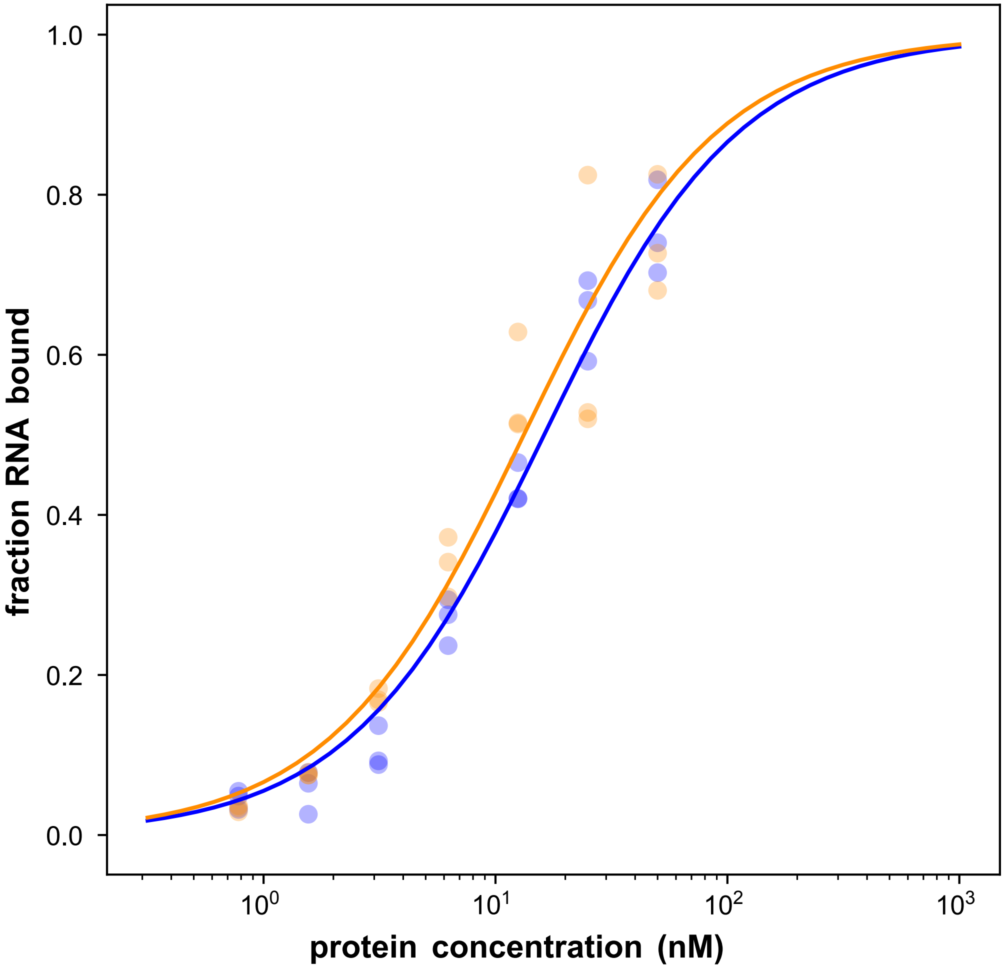

Supplementary Figure S5. Binding of 9S arrays to *rpoA* oligonucleotides using either unpurified bacterial lysate (blue) or purified protein (orange). Binding was measured by RNA pulldown. The proteins tested were GFP-9S in both cases. The apparent K_d values estimated from the fitted curves were 15 nM and 12 nM respectively. The curve-fit equation used was equation (3) from Pagano et al. RNA (2011), 17:14–20.

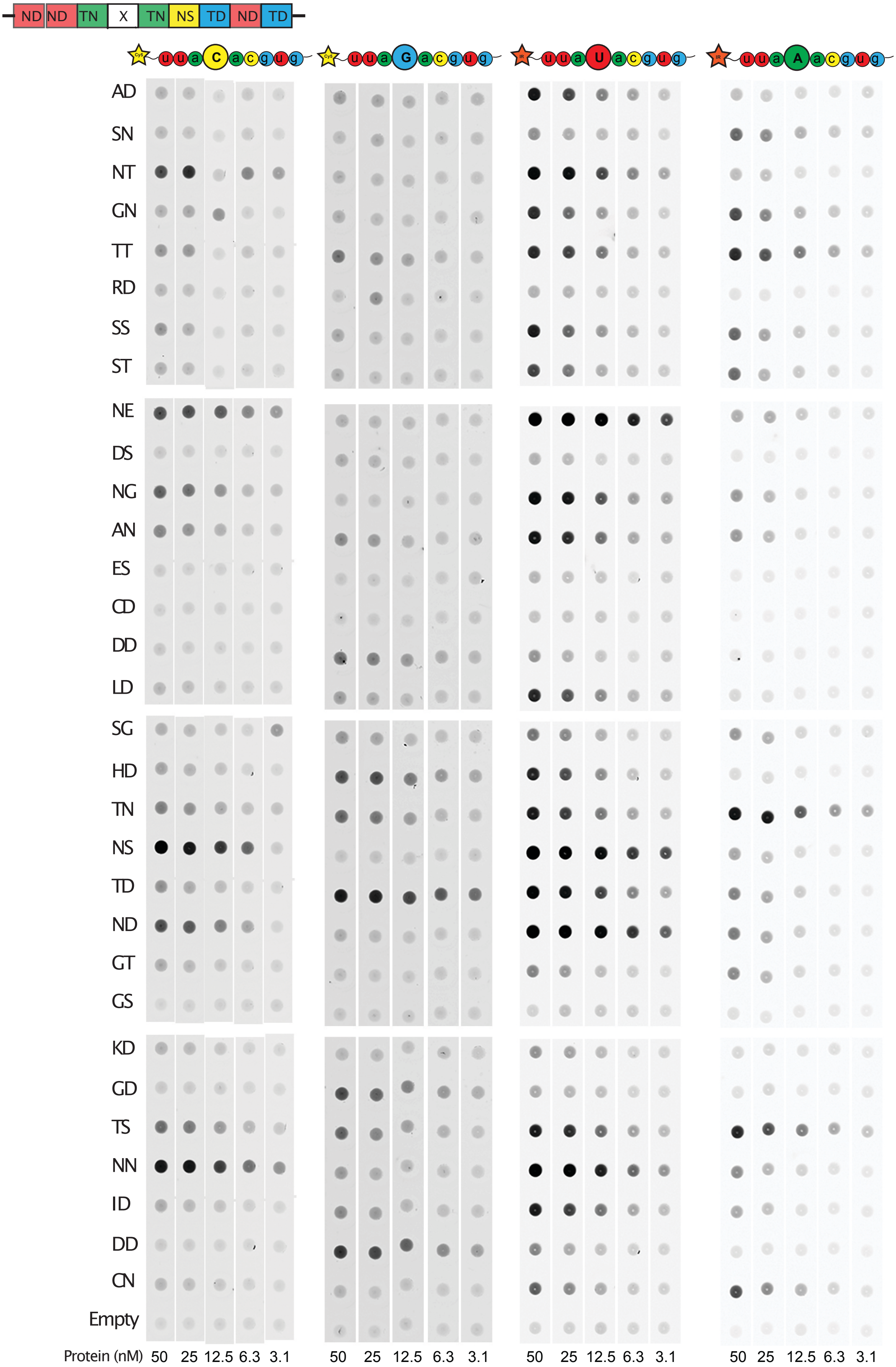

Supplementary Figure S6. Screen of binding specificity of selected 5th/last positions in S-type PPR motifs. Each GFP-9S variant was tested at 5 different concentrations against 4 RNA targets differing by a single nucleotide. Binding was measured by RNA pulldown; each spot shows the fluorescence signal from the bound and eluted RNA. This experiment was repeated three times; the figure shows a single representative experiment.

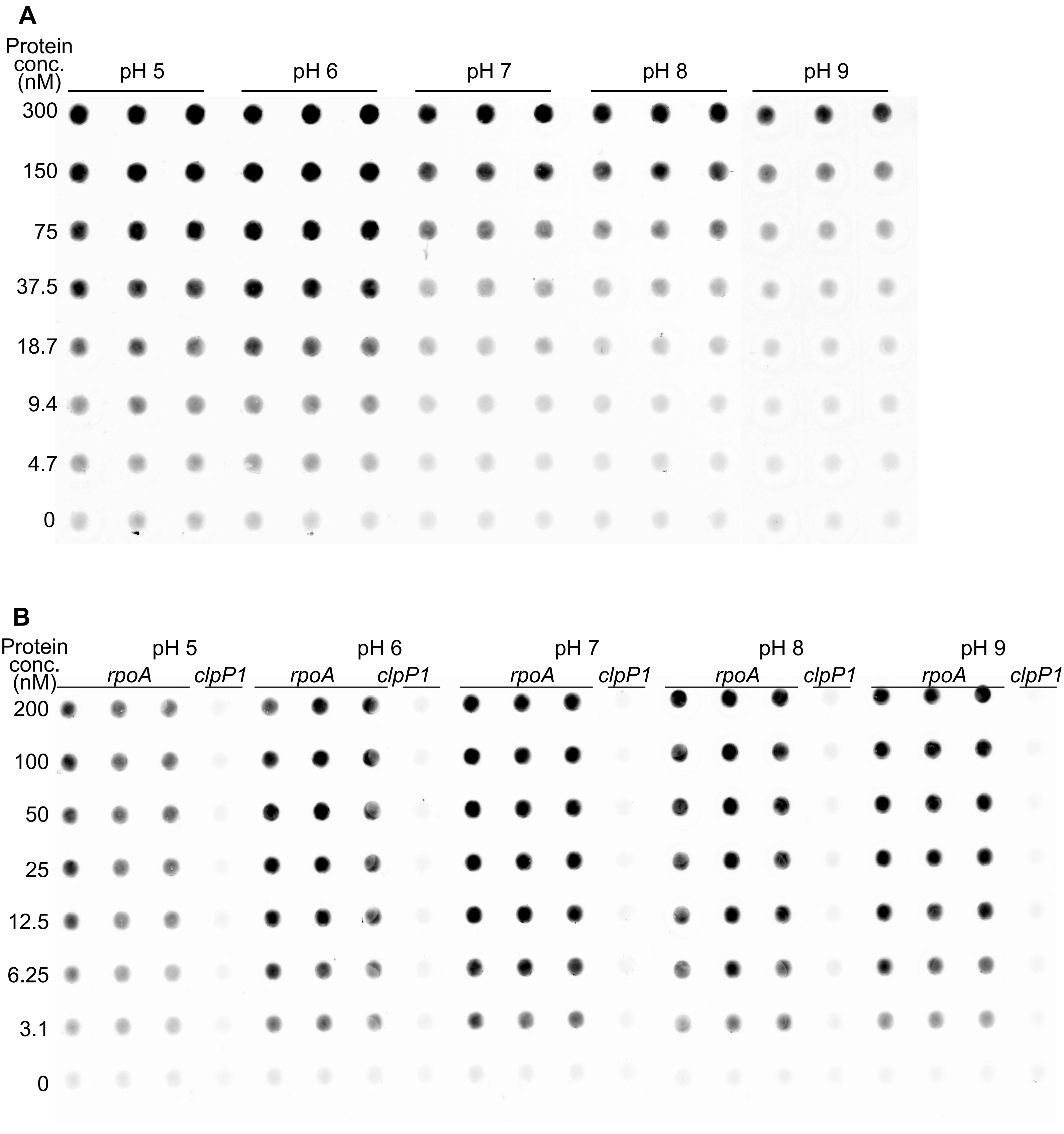

Supplementary Figure S7. Binding of (**A**) TRX-9P-S2 and (**B**) TRX-9S-S2 to *rpoA* and *clpP1* target probes at various pH values.

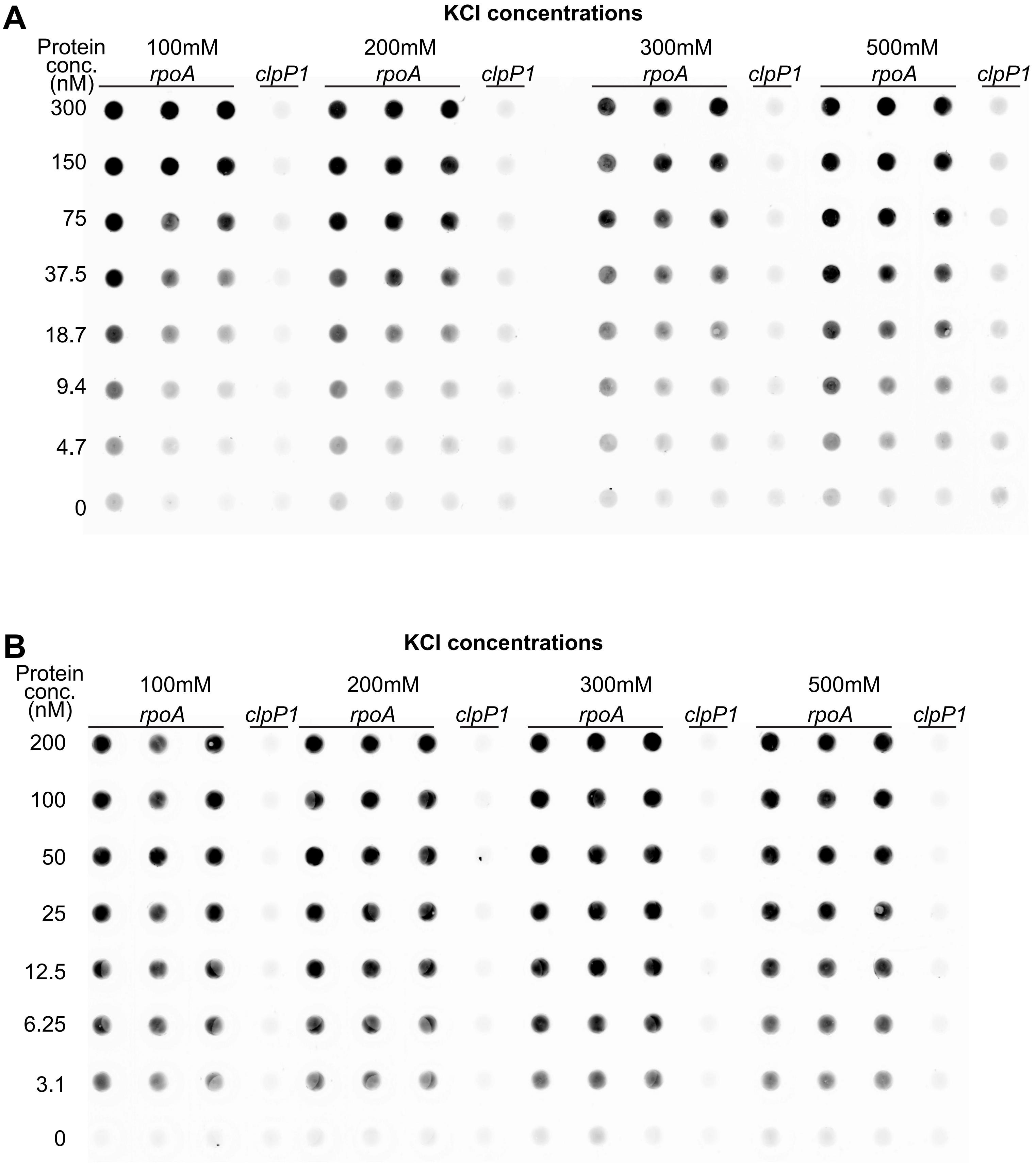

Supplementary Figure S8. Binding of (**A**) TRX-9S-S2 and (**B**) TRX-9P-S2 to *rpoA* and *clpP1* target RNAs at various KCl concentrations at pH 8. These are the data for the plots in Figure 4B.

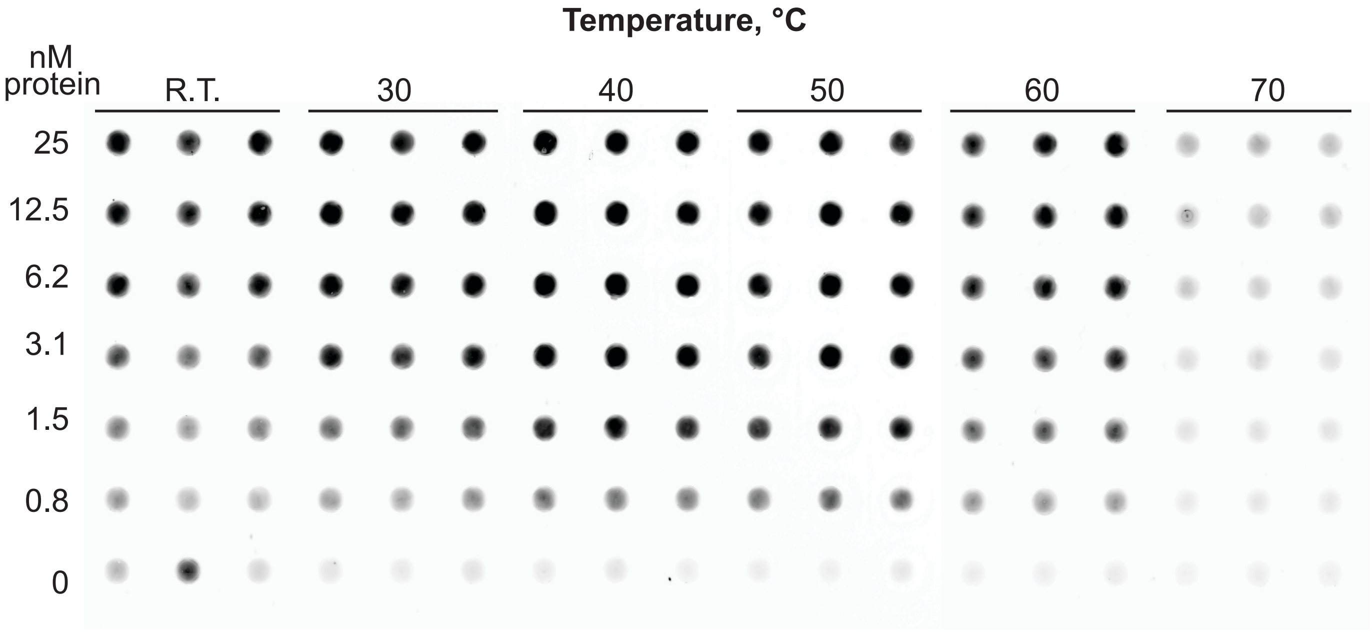

Supplementary Figure S9. Biotinylated TRX-AviTag-9S-S2 binding to *rpoA* RNA at different temperatures. R.T., room temperature. The measurements were done at pH 7 and 150 mM KCl. These are the data for the plot in Figure 4C.
